# Entomological baseline data collection and power analyses in preparation of a mosquito swarm-killing intervention in south-western Burkina Faso

**DOI:** 10.1186/s12936-021-03877-x

**Published:** 2021-08-23

**Authors:** Abdoulaye Niang, Simon P. Sawadogo, Abdoul A. Millogo, Nwamaka O. Akpodiete, Roch K. Dabiré, Frederic Tripet, Abdoulaye Diabaté

**Affiliations:** 1 Institut de Recherche en Sciences de la Santé, Bobo-Dioulasso, Burkina Faso; 2 Institut des Sciences des Sociétés (INSS), Ouagadougou, Burkina Faso; 3 Centre for Applied Entomology and Parasitology, School of Life Sciences, Keele University, Staffordshire, UK

**Keywords:** Baseline data, Malaria vector control, Power analyses, Swarm-killing intervention, Burkina Faso

## Abstract

**Background:**

Insecticides are currently the main tools used to reduce the transmission of malaria; therefore, the development of resistance to insecticides in malaria vectors is of major concern for malaria control. The resistance level to pyrethroids is particularly high in the Western region of Burkina Faso and may affect the efficacy of insecticidal bed nets and indoor residual spraying. Adult mosquito swarming and other nocturnal behaviours exhibit spatial and temporal patterns that suggest potential vulnerability to targeted space spraying with effective insecticides. Indeed, targeted space-spraying against adult mosquito swarms has been used to crash mosquito populations and disrupt malaria transmission.

**Methods:**

Prior to impact assessment of swarm killing, a baseline data collection was conducted from June to November 2016 in 10 villages divided into two areas in western Burkina Faso. The data considered both ecological and demographic characteristics to monitor the key entomological parameters.

**Results:**

The mean number of swarms observed was 35 per village, ranging from 25 to 70 swarms according to the village. Female density in both areas varied significantly as a function of the village and the period of collection. The human biting rate was significantly affected by the period of collection and depended upon whether the collection was carried out indoors or outdoors. Averages of parity rate were high in both areas for all periods of collection, ranging from 60 to 90%. These values ranged from 80 to 100% for inseminated females. Sporozoite rates ranged between 1.6 and 7.2% depending upon the village. The molecular identification of resting and swarming mosquitoes showed the presence of the three major malaria vectors in Burkina Faso, but in different proportions for each village.

**Conclusions:**

The distribution of the potential swarm markers and swarms in villages suggested that swarms are clustered across space, making intervention easier. Power simulations showed that the direct sampling of swarms provides the highest statistical power, thereby reducing the number of villages needed for a trial.

**Supplementary Information:**

The online version contains supplementary material available at 10.1186/s12936-021-03877-x.

## Background

Since 2000, huge efforts have taken place to fight neglected tropical diseases, but malaria remains the principal cause of death and represents a major public health problem in the African continent. More than 3.2 billion people are at risk of contracting malaria around the world, accounting for over 228 million clinical cases specifically in the tropical regions including South America, Southeast Asia and sub-Saharan Africa [1]. Malaria prevalence is high with over 400,000 deaths per year around the world. Approximately 60 and 15% of deaths recorded were children below 5 years of age and pregnant women respectively. Ninety-two per cent of these deaths were in the sub-Saharan Africa region [1]. In Burkina Faso 11 million malaria cases were reported in 2018 with 4294 resulting deaths [2]. Long-lasting insecticidal nets (LLINs) and indoor residual spraying (IRS) are the two front-line strategies recommended to interrupt malaria transmission [3, 4]. These strategies have contributed to a reduction of malaria transmission in several areas of the world and are particularly dependent on the biting and resting behaviour of mosquito vectors [5–9]. In Burkina Faso, mass distribution campaigns in 2010, 2013 and 2016 distributed approximately 36 LLINs as the primarily method of malaria prevention. In 2010 an IRS pilot programme using bendiocarb, a carbamate insecticide, was introduced in the Diébougou district of the southwestern region of Burkina Faso (http://www.africairs.net/where-we-work/burkina-faso/). Although interrupted for many years, the IRS programme was re-started in the same district with some innovations.

Within the last two decades many studies have reported insecticide resistance spreading to all the vector species throughout Africa [10–15]. The development of resistance to insecticides in malaria vectors is the main concern for malaria control as currently major vector control tools rely on the use of insecticide. Resistance to the four major classes of insecticides (organochlorides, organophosphates, pyrethroids, carbamates) that are used in public health has recently increased throughout Burkina Faso. The resistance level to pyrethroids is particularly high in the Western region [16–18], which may affect the efficacy of LLINs and IRS.

In Burkina Faso, *Anopheles gambiae*, *Anopheles coluzzii*, *Anopheles arabiensis* and *Anopheles funestus* are the main malaria vectors [1, 16, 18–20]. Members of the *An. gambiae* complex are found to be sympatric in several localities of the country with different ecological niches [16, 21–23]. *Anopheles gambiae* is present in all regions of the country, *An. arabiensis* is more abundant in the Sudano-Sahelian zone and Sahelian zone. *Anopheles coluzzii* is more frequent in the rice-growing zone in the western part of the country [18, 22]. *Anopheles funestus* is more abundant in the Sudanian zone and its abundance decreases towards the Sahelian zone [16, 22, 24].

Several studies have shown that malaria infection is influenced by environmental factors, such as temperature, precipitation and relative humidity, which vary from region to region [14, 24]. However, in most parts of Africa, there is still a lack of information regarding the dynamics of vector population and malaria transmission that can guide the implementation of vector control interventions [25, 26]. Additionally, malaria transmission in Africa will be difficult to control unless novel methods are developed to suppress residual outdoor transmission. Mosquitoes that bite people outdoors and also rest outdoors generally avoid the indoor interventions such as LLINs and IRS. The use of targeted space spraying against adult mosquito swarms and other nocturnal *Anopheles* behaviours has been designed to crash populations of these mosquitoes and disrupt malaria transmission. Adult mosquito swarming and other nocturnal behaviours such as host seeking, oviposition site seeking, sugar feeding, and resting, all exhibit spatial and temporal patterns that suggest potential vulnerability to targeted space spraying with effective insecticides. In a recent preliminary study conducted in VK5 in western Burkina Faso to assess the impact of swarm control, repeated target swarm killing with bomb spray containing a mixture of pyrethroid and carbamate resulted in mosquito density reduction [27]. Interestingly, that intervention has clearly affected the age structure of the population, which was strongly shifted towards immature males and the female insemination rate significantly decreased. However, while the effectiveness of the approach against *An. coluzzii* has been proven in that small scale of the study village, it needs to be broadened and validated for other vector species. In addition, the efficacy of this approach and that of swarm killing by broadcast space spraying has not been formally tested on a large scale in Africa.

In this study it was proposed to use the WHO Pesticide Evaluation Scheme (WHOPES) recommended insecticides, sprayed at dusk by trained community-based spray teams using conventional broadcast spraying by back-pack sprayers to achieve maximum effect. Prior to intervention and impact assessment of this approach, a sampling strategy was defined to consider both ecological and demographic characteristics in the estimation of key baseline entomological parameters. These included the relative abundance of adult mosquito populations both indoors and outdoors, swarming behaviour, adult female insemination status, age structure, and mosquito biting rates. Aside from reporting on the ecology of vectors and malaria transmission in a large study area with different ecological settings in the western Burkina Faso, it was decided to use the baseline data to simulate the power of randomized trial, thereby informing the design and methodologies of the upcoming intervention.

## Methods

### Study areas

**Fig. 1:**
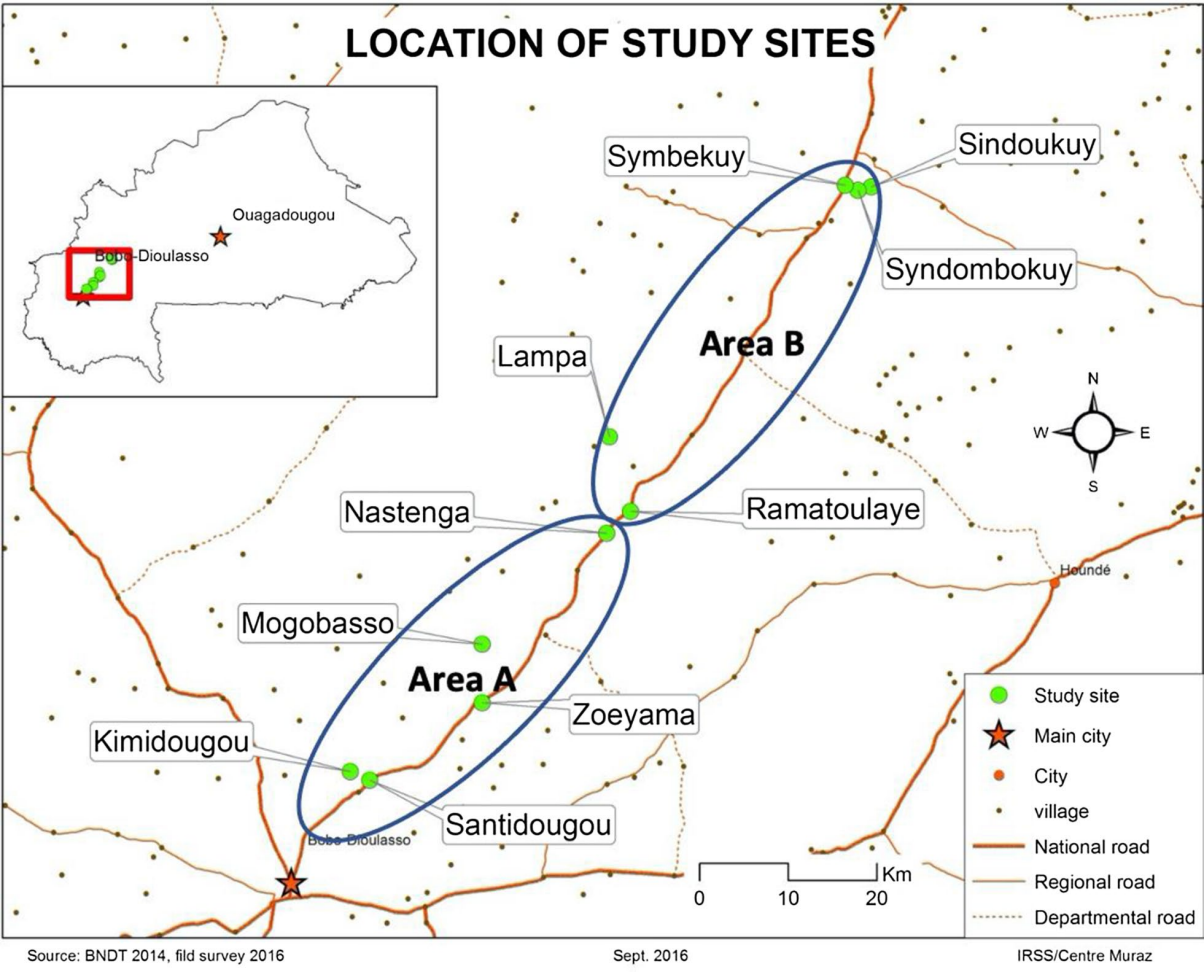
Location of the study sites within Areas A and B in the southwestern region of Burkina Faso

The study was conducted in 10 villages located in the western region of Burkina Faso. The study sites were situated along the National Road 10 over 105 km, in the humid savannah eco-epidemiological zone. The villages were: Santidougou, Kimidougou, Nastenga, Zeyama, Mogobasso, and Ramatoulaye in the district of Lena; Synbekuy, Syndombokuy, Lampa, and Syndounkuy in the district of Dedougou. The villages were then assigned to an area, each area containing five villages: Area A (Santidougou, Kimidougou, Nastenga, Zeyama, Mogobasso) and Area B (Synbekuy, Ramatoulaye, Syndombokuy, Lampa, Syndounkuy) (Fig. 1). Both areas have two distinct annual seasons: a short rainy season from May to October with a peak in August to September and a long dry season from November to April. Average annual rainfall ranges from 1000 to 1200 mm, which allows the presence of temporary and semi-permanent pools suitable for mosquito larval development. All selected villages lie in the cotton belt of Burkina Faso, where insecticides against agricultural insect pests are used intensively during the cropping period. The accessibility of the study sites in both seasons, the size of the population and the distance from the surrounding villages were considered when they were selected.

### Swarm characterization

In each of the 10 study sites the anopheline swarms were monitored by trained observers during 6 months from June to November 2016; this period overlapped with the main malaria transmission season in the study areas. The potential swarm markers and concessions were identified and geo-referenced using a global positioning system (GPS) GARMIN, series GPSMAP®62.2.3 with measurements of latitude and longitude accurate to within 3 m. The concessions were labelled with a unique number for the whole study period using paint. In anopheline mosquitoes mating swarms are free-flying aggregations of males, which form at dusk. Females searching for a mate approach and enter these swarms, leading to the formation of mating pairs that fall down or fly out the swarm in copula. Males of the major malaria vectors predictably use the same sites, exploiting distinctive landmarks to gather and mate, and the same locations are used for swarming over several years [28, 29]. Swarms were then sampled four times every month from June to November using insect nets as described previously [28, 30, 31]. Swarm sites of *An. gambiae* sensu lato (s.l.) (see Additional files 1 and 2) were observed above physical markers scattered throughout the villages. The physical makers comprised waste, manure heaps, wood piles, wells, walls, grass, toilets, and patches of bare ground. The nature of the swarm marker, swarm duration and the number of swarming males were recorded. Mosquitoes were aspirated into separate cups for each swarm, killed with ethylic ether, identified morphologically as *An. gambiae* s.l. [32, 33] and kept in 70% ethanol in 1.5 ml tubes.

### Mosquito collections

During the same period as the swarm monitoring, anopheline mosquitoes were collected during the rainy season from June to November 2016 by two sampling methods: the pyrethrum spray catch (PSC) and the human landing catches (HLC) [34]. Resting mosquitoes were caught by spraying houses or inhabited huts in the 10 villages with insecticide aerosols. In each village, a total of 20 houses were chosen for mosquito collection, 10 selected randomly each month, and 10 fixed during the whole period of the study. One collection session was performed from 07.00 to 09.00 h per month over the 6 months. A PSC of Kaltox® was used and white sheets laid on the floor. Knocked down mosquitoes fell onto the white sheets and were immediately retrieved. This sampling method gave an accurate estimation of the total density of mosquito species in the houses (see Additional file 3 for composition of the culicidae fauna in the villages in Areas A and B). HLCs were performed by trained volunteers who were provided with free and rapid treatment if they displayed clinical signs of malaria (as defined by the World Health Organization (WHO) recommended regimen based on fever and detectable *Plasmodium falciparum* parasitaemia). To evaluate human biting rates (HBR), pairs of adult human males sat indoors and outdoors collecting the mosquitoes that landed on them by means of a flashlight and glass tubes. Collections were carried out between 20:00 and 06:00 inside and outside of four houses in each village. To standardize catching efficiency, collectors rotated between houses on subsequent nights. Female and males of the *An. gambiae* complex were identified morphologically as described above [32, 33]. A sample of females was dissected, and the head and thorax preserved to determine the infectious status. Legs were separated from the carcass and kept dry for molecular species identification. Spermathecae were removed and dissected to determine their insemination status as described previously [31]. Ovaries were processed likewise to determine the population age structure using the ovary tracheation method, the most applied morphological age classification technique for mosquitoes [35].

### Laboratory processing of mosquitoes

The head and thorax of females belonging to the *An. gambiae* complex (identified according to the standard identification keys [32, 33]) were removed and tested by enzyme-linked immunosorbent assay (ELISA) [36] to determine the presence of the circumsporozoite protein (CSP) of *P. falciparum*, the major malarial parasite occurring in most of the African region countries, including Burkina Faso. Two random samples including a cohort of females tested by ELISA and a sample of males from the swarms were processed by PCR for molecular identification at the species level [37].

### Statistical analysis

The resting mosquito abundance (number of mosquitoes), the HBR (number of bites per person per night), the parity rate (percentage of parous females), and the insemination rate (percentage of inseminated females) were defined as the key entomological parameters to determine the dynamic of *An. gambiae* s.l. populations and that of the malaria transmission in the different study sites of both areas. Analyses were carried out in R version 3.5.2 using the ‘lme4’ package. The general linear model (glmmTMB function) was used to test whether the number of resting mosquitoes per house and the HBR varied with the locality (village) and the period (month) of collection. The logistic regression model was used to test the effect of the locality and the period of collection on the parity rate and the insemination rate of females, respectively. For all models, the residuals were plotted to check for homoscedasticity, independence and normality and, where appropriate, *post-hoc* Tukey tests were carried out to make pairwise comparisons.

Cluster randomized intervention trial (CRT), non-aggregated power analyses were performed using the approach described in Gao et al. [38]. Assuming a control and an intervention arm, the analysis used the mean and standard deviations of mosquito abundance collected via swarm captures, PSC and HLC and the same sampling effort used in the baseline studies to estimate the number of villages required to achieve a given statistical power as a function of varying projected intervention impacts (percent population reductions).

## Results

### Swarm characterization

Swarms of *An. gambiae* s.l. were observed above miscellaneous physical markers such as waste, manure heaps, wood piles, wells, walls, grass, toilets, and above bare ground. Swarms of *Culex* were often observed in the villages. The mean number of swarms observed was 35 per village and ranged from 25 to 70 swarms according to the village (one-way analysis of variance, *P* = 0.25). The size of observed swarms varied between 5 and 150 mosquitoes with a mean of 50 mosquitoes per swarm and it was varied significantly between the villages (one-way analysis of variance, *P* < 0.05). The low number of mosquitoes in most swarms indicated that it was not necessary to use a high quantity of insecticide to kill mosquitoes and to stop mating. The mean height above the ground was 2 m and varied significantly between the study villages (one-way analysis of variance, *P* < 0.05). The distribution of potential swarm markers and observed swarms in the village level, as shown in Additional files 1 and 2 for Area A and Area B, respectively, suggests that swarms were clustered across space, making the intervention easier. The interaction of mosquitoes with the potential swarm markers and other elements in the compound, such as trees and houses, shows that swarms are located inside the boundaries of the villages as they use man-made swarm markers.

### Resting mosquito density

**Fig. 2:**
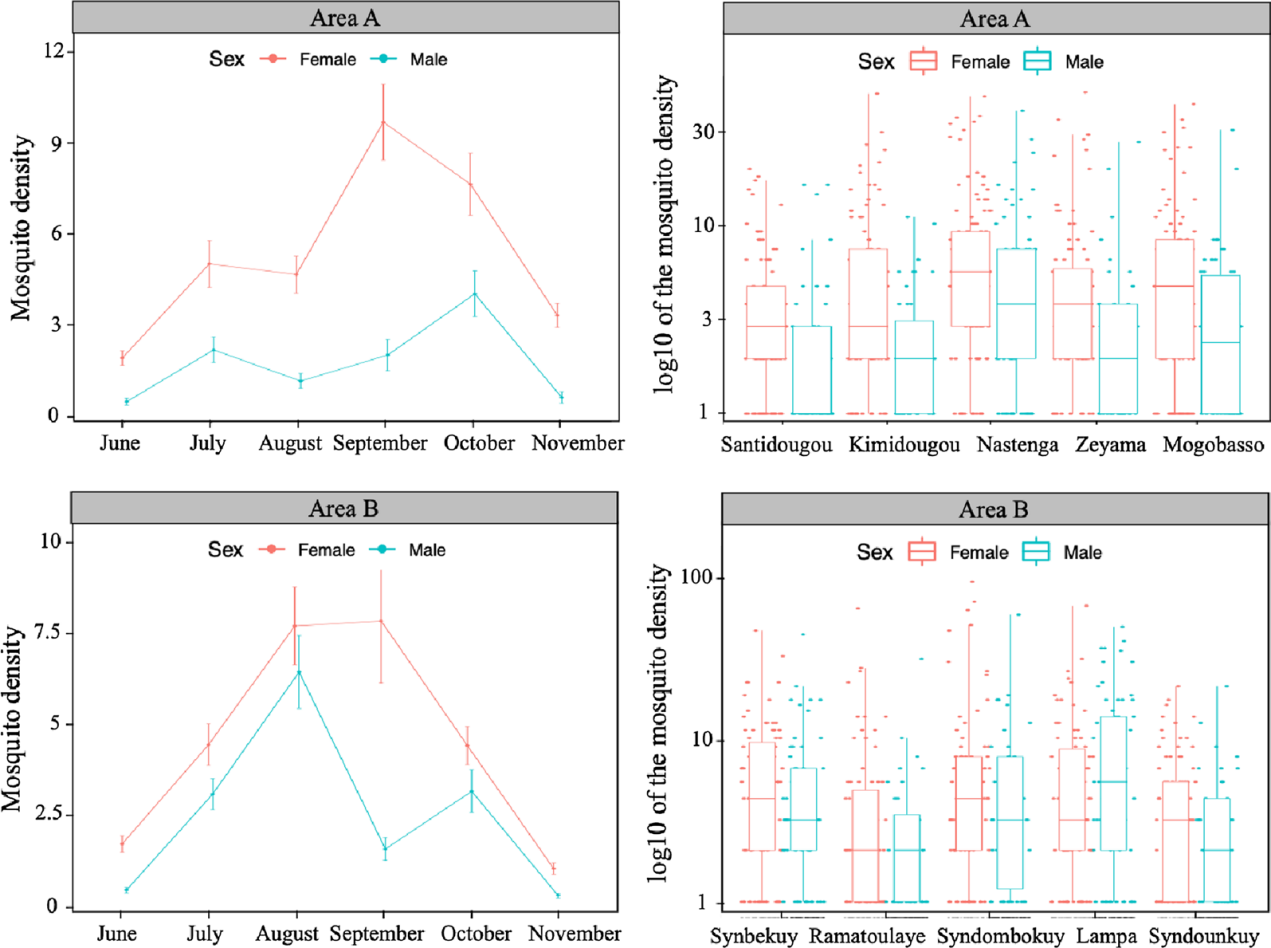
Resting mosquito abundance: distribution of the number of males and females per house in the villages of Areas A and B during 6 months of collection

Resting mosquito abundance was estimated monthly by calculating the mean number of mosquitoes per house for a period of 6 months in the villages of Areas A and B. A total of 8281 samples was collected PSC, of which 5802 (70.06%) were females and 2479 (29.94%) males. The result showed significant variation between sex (*χ*^*2*^ = 185.94; *df* = 1; *P* < 0.001). The mean density was two-fold higher for females with 4.83 mosquitoes per house; the mean density for males was 2.07 mosquitoes per house. However, variations of the mosquito density according to the area (*χ*^*2*^ = 0.29; *df* = 1; *P* = 0.594) and the period (*χ*^*2*^ = 2.86; *df* = 1; *P* = 0.09) of collection were not significant. The interaction between sex and area (*χ*^*2*^ = 10.34; *df* = 1; *P* = 0.001) and that between period and area of collection (*χ*^*2*^ = 15.32; *df* = 1; *P* < 0.001) were significant, while no significant interaction was found between sex and period of collection (*χ*^*2*^ = 0.27; *df* = 1; *P* = 0.602). Similarly, the triple interaction between sex, area and period of collection was not significant (*χ*^*2*^ = 0.25; *df* = 1; *P* = 0.618). To check how the mosquito resting abundance varied between villages and periods of collection within the two areas, data from the females were used as the response variable for the subsequent model because the numbers of males and females per house were highly correlated (Pearson’s correlation, r = 0.94, *t* = 18.666, *df* = 1198, *P* < 0.001). In total, 3323 females were collected in Area A with 5.54 females per house, while 2479 females were collected in Area B with a mean of 4.13 females per house. In Area A the female density varied significantly as a function of the village (*χ*^*2*^ = 27.54; *df* = 4; *P* < 0.001) and the period (*χ*^*2*^ = 86.56; *df* = 5; *P* < 0.001) of collection as well. The interaction between village and period of collection was also significant (*χ*^*2*^ = 51.25; *df* = 20; *P* < 0.001). Female abundance was higher in Nastenga followed by Mogobasso, Kimidougou and Zeyama with the mean densities of 7.92, 6.05, 5.26, and 5.19 mosquitoes per house, respectively, while the lowest density was found in Santidougou with 2.27 females per house (Fig. 2). In this area according to the period of collection, the highest densities were sampled in September and October while the lowest density was recorded in June (Fig. 2). Similarly, in Area B the variation of the female density was significant according to the village (*χ*^*2*^ = 22.64; *df* = 4; *P* < 0.001) and the period (*χ*^*2*^ = 137.3; *df* = 5; *P* < 0.001) of collection. The interaction between village and period of collection was also significant (*χ*^*2*^ = 74.73; *df* = 20; *P* < 0.001). Female density was higher in Syndombokuy followed by Synbekuy and Lampa with the mean densities of 6.88, 4.23 and 3.86 mosquitoes per house, respectively, while the lowest densities were found in Syndounkuy and Ramatoulaye with 2.87 and 2.82 females per house, respectively (Fig. 2). In this area, according to the period of collection, the highest densities were sampled in August and September while the lowest density was recorded in November (Fig. 2).

### Human biting rate (HBR)

**Fig. 3:**
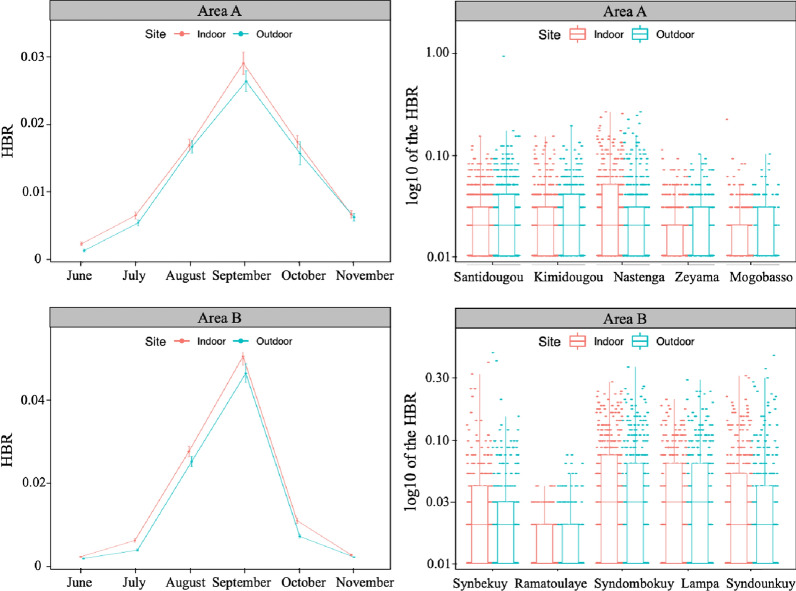
Human biting rate: distribution of number of bites per person per night indoors and outdoors in the villages of Areas A and B

The HBR was calculated as the number of mosquito bites per person per night in the 10 villages of collection divided into two areas, A and B, over 6 months from June to November 2016 (Fig. 3). In total 21,546 females of *An. gambiae* s.l. were collected with an average of 11.22 bites/person/night. The HBR was significantly affected by the period of collection (*χ*^*2*^ = 5,00.6; *df* = 5; *P* < 0.001) and by the fact that collection was carried out indoors or outdoors (*χ*^*2*^ = 13.89; *df* = 1; *P* < 0.001), but it was similar in the two areas, A and B (*χ*^*2*^ = 0.22; *df* = 1; *P* = 0.635). The HBR was higher indoors than outdoors (Fig. 3) with an average of 11.95 bites/person/night and 10.52 bites/person/night, respectively. According to the period of collection, it was higher from August to October compared to June/July and November; the highest mean values were recorded in September for both areas (Fig. 3). Taken in pairs, the double interactions area: period (*χ*^*2*^ = 942.57; *df* = 5; *P* < 0.001) and the site: period (*χ*^*2*^ = 38.11; *df* = 5; *P* < 0.001) were significant, whereas the interaction area: site was not significant. The triple interaction site: period: area was significant (*χ*^*2*^ = 23.72; *df* = 5; *P* < 0.001). A more detailed analysis was carried out to assess the influence of the village and the period of collection on the mosquito bite in each of the two areas. In Area A the HBR was significantly affected by the village (*χ*^*2*^ = 538.66; *df* = 4; *P* < 0.001) and the period of collection (*χ*^*2*^ = 1603.4; *df* = 5; *P* < 0.001). Higher HBR mean values were recorded in Santidougou, Kimidougou and Nastenga compared to those of the remaining villages of Mogobasso and Zeyama (Fig. 3). The interaction between village and period was significant as well (*χ*^*2*^ = 640.64; *df* = 20; *P* < 0.001). The result was similar in the Area B with a significant influence of the village (*χ*^*2*^ = 4464.33; *df* = 4; *P* < 0.001), the period (*χ*^*2*^ = 4377; *df* = 5; *P* < 0.001) and their interaction (*χ*^*2*^ = 222.43; *df* = 20; *P* < 0.001). Here, higher HBR mean values were recorded in Syndombokuy and Lampa followed by Synbekuy and Syndounkuy. Ramatoulaye scored the lowest value (Fig. 3).

### Parity rate and insemination rate

**Fig. 4:**
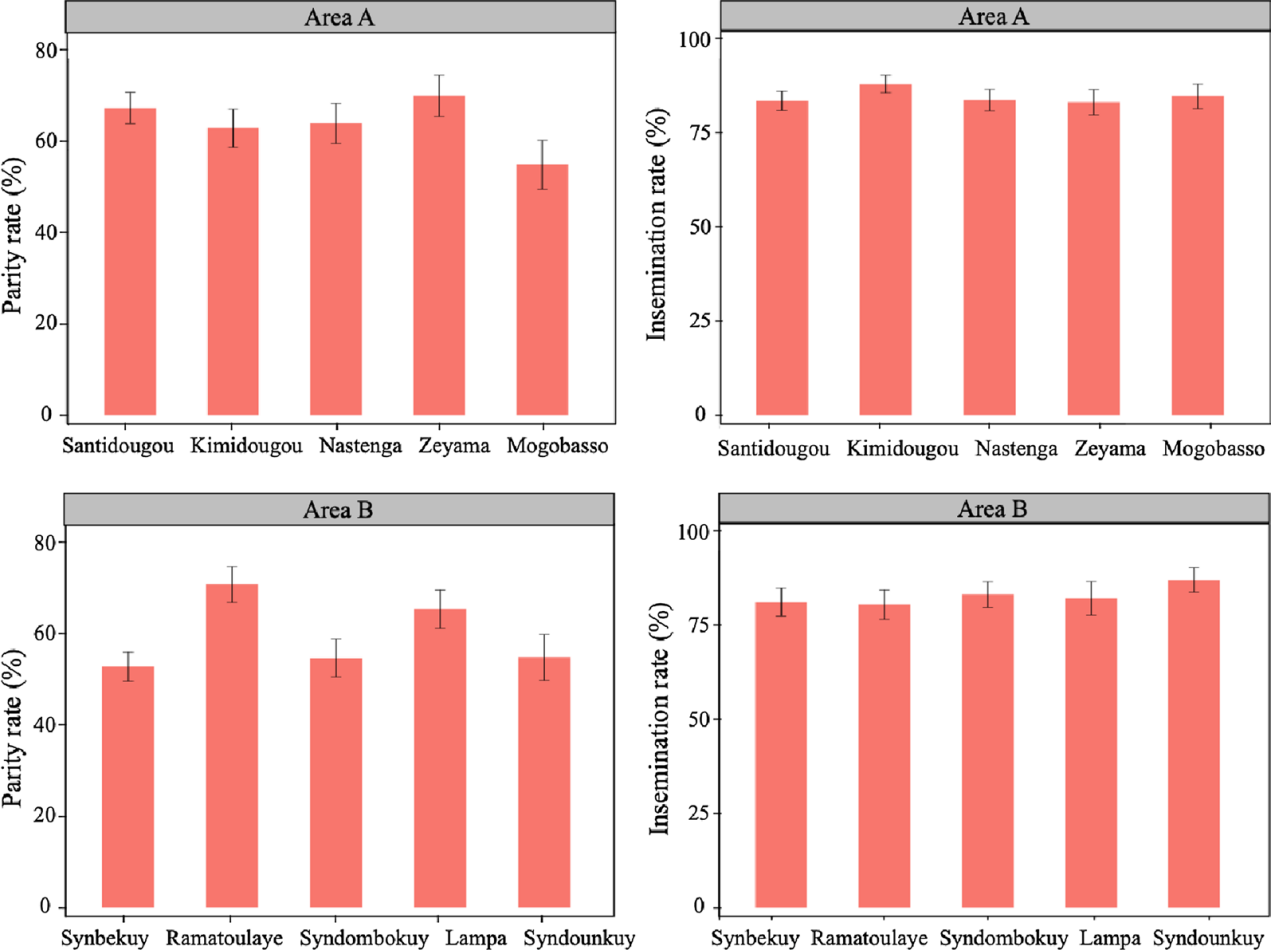
Proportion of the inseminated and parous females collected in the villages of Areas A and B

In total, 4195 females were tested for the parity rate and the insemination calculation as the proportion of the parous and the inseminated females, respectively. The parity status of 316 females and insemination status of 533 females remained undetermined. These females were removed from the analyses. The averages of the parity rate were high in the 10 villages during the periods of collection ranging from 60 to 90% (Fig. 4). Similarly, from 80 to 100% of females were found to be inseminated (Fig. 4). The parity and the insemination rates of females collected monthly during June to November from villages within Areas A and B were analysed separately using logistic regression model (Table 1). In both areas the period of collection strongly affected the parity rate (*P* < 0.001). In Area A there was no direct significant effect of the village on parity rates (*P* = 0.724), but a significant interaction between the period and the village of collection on parity rates (*P* < 0.029) (Table 1). Similarly, in Area B parity rates were not directly affected by the village of collection (*P* = 0.724), but there was a strong and significant interaction between the period and the village of collection on parity rates (*P* < 0.001) (Table 2). The parity rate was significantly higher in October (*P* = 0.015) in Area A, while this was true in August for Area B (*P* = 0.038) (Table 2).

**Table 1:** Logistic regression (effect likelihood ratio tests) of the effects of period and village of collection on female parity rates in the Areas A and B

Area	Source	Df	L-ratio	P-value
Area A	Village	4	2.06	0.724
	Month	5	135.96	< 0.001*
	Village * month	16	28.29	0.029
Area B	Village	4	6.12	0.190
	Month	4	31.35	< 0.001*
	Village * Month	15	59.24	< 0.001*

**Table 2:** Logistic regression (effect likelihood ratio tests) of the effects of period and village of collection on female insemination rates in the Areas A and B

Area	Source	Df	L-ratio	P-value
Area A	Village	4	3.09	0.543
	Month	5	26.89	< 0.001*
	Village * month	13	17.42	0.181
Area B	Village	4	8.26	0.083
	Month	3	29.85	< 0.001*
	Village * month	11	26.58	0.005*

In addition, the insemination rate did not significantly vary between the villages in Area A (*P* = 0.543) nor in Area B (*P* = 0.083) but it was significantly affected by the period of collection in both areas (*P* < 0.001). The interaction between the period and the village of collection on insemination rates was significant in Area B (*P* = 0.005) but not in Area A (*P* = 0.181).

### Sporozoite indices and entomological inoculation rates

In total, 4001 mosquitoes were tested by ELISA-CSP. The sporozoite rates ranged between 1.6 and 7.2% depending on the villages; the highest value was reported in Nastenga. The annual entomological inoculation rates (EIR) varied greatly depending on the village as well (0.2 to 1.1 infective bite/person/night) and transmission was particularly high in Nastenga (Table 3).

**Table 3:** Infection and entomological inoculation rate in the villages of the Areas A and B in western Burkina faso

Area	Village	SI (%)	EIR (IB/P/N)
Area A	Santidougou	5.90	0.60
	Kimidougou	5.90	0.90
	Nastenga	7.20	1.10
	Zeyama	5.30	0.40
	Mogobasso	2.80	0.15
Area B	Synbekuy	3.50	0.30
	Ramatoulaye	6.50	0.20
	Syndombokuy	1.60	0.30
	Lampa	4.00	0.40
	Syndounkuy	4.60	0.70

IB/P/N: infective bite per person per night

### Specific composition of *Anopheles gambiae* s.l.

**Fig. 5:**
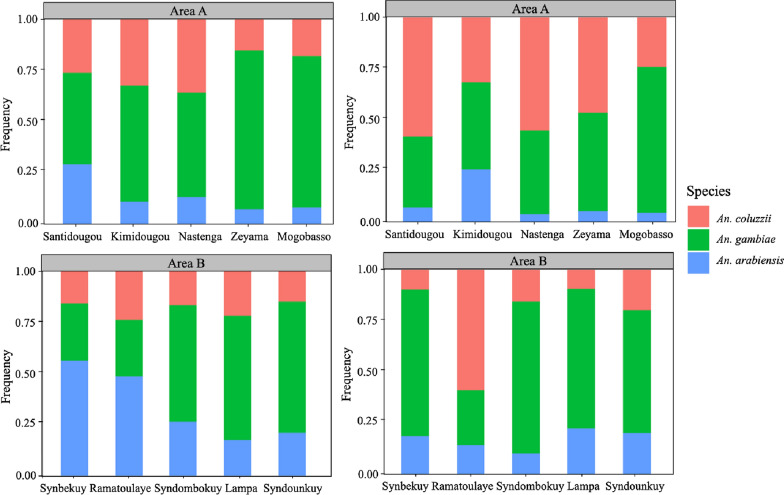
Proportions of resting mosquitoes and swarming males for three *Anopheles gambiae* complex species in the villages of Areas A and B

In total, 1784 resting mosquitoes were analysed by PCR and the result showed the presence of the three major malaria vectors in Burkina Faso: *An. arabiensis*, *An. gambiae* and *An. coluzzii* in different proportions (Fig. 5). Globally *An. gambiae* was the most represented with 52.4%, followed by *An. arabiensis* (24.3%) and *An. coluzzii* (23.3%). A total of 430 males from the swarms analysed by PCR showed the presence of the three species listed above with 48, 41 and 11% for *An. gambiae*, *An. coluzzii* and *An. arabiensis*, respectively (Fig. 5).

### Predicted cluster randomized trial power

**Fig. 6:**
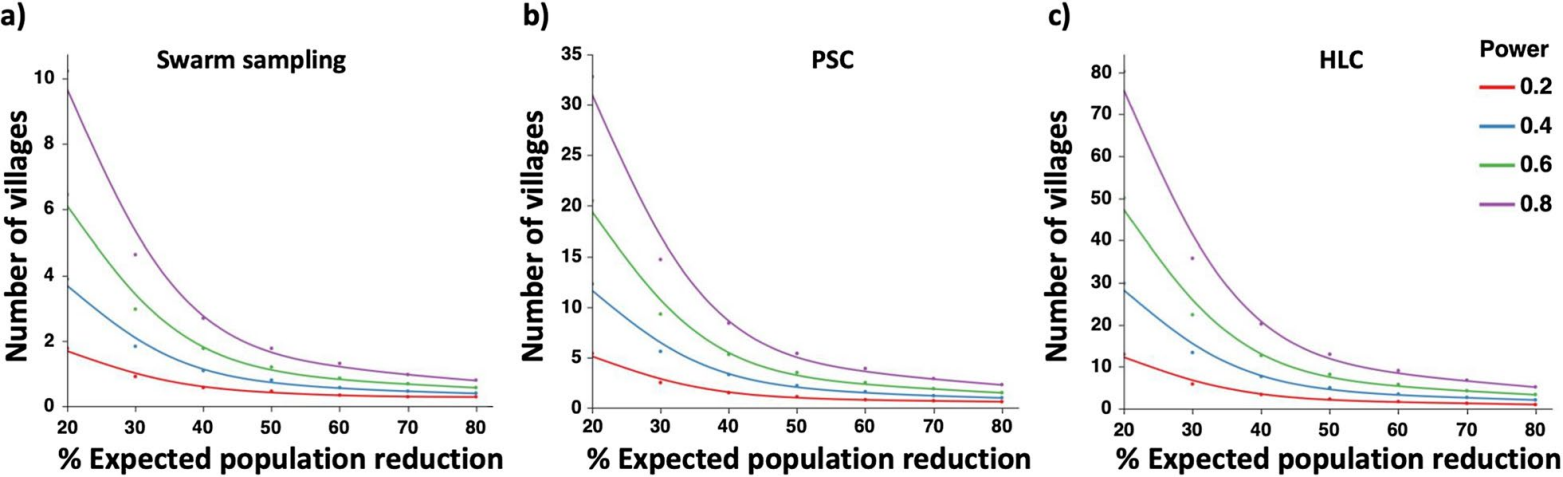
Cluster-randomized trial (CRT) power simulations showing the number of villages needed per arm (control or intervention) in relation to the percentage mosquito population reduction expected; simulations assumed a sampling effort similar to that undertake to establish the baseline data

Statistical power simulations using varying predicted intervention impacts were performed to assess how much power could be achieved using the 10 villages surveyed (Fig. 6a–c). The results showed that provided the same sampling efforts were made for the CRT as in baseline studies, PSC and HLC would result in vastly underpowered statistical comparisons unless the expected intervention impact would be high. For PSC, adequate power (0.6 and 0.8 or higher) could be achieved assuming a mosquito density reduction equivalent of higher than 30 and 40%, respectively (Fig. 6b, c). For densities measured through HLC, adequate power could only be achieved assuming intervention impacts equivalent to 70% population reduction or higher, suggesting that sampling effort should be increased under any scenarios. Importantly, given the focus of the planned swarm control intervention, the high mean number of swarms collected (n = 35) provided statistical power equivalent to 0.8 or higher even when assuming a low impact of the intervention (20%) (Fig. 6a).

## Discussion

This study showed that swarms were located within the villages of Areas A and B and clustered in specific locations depending on the availability of markers, confirming the previous study in Burkina Faso [29–31, 39]. The average number of swarms observed was 35 per village, which suggested that in the typical savannah of Sudan the number of swarms would not be too high and could easily be covered in one day of intervention. Depending on the markers, the 35 swarms could be distributed in only 10 to 15 compounds and one volunteer would be able to monitor an entire compound. It was observed that the size of swarms averaged 50 mosquitoes per swarm. The low number of mosquitoes in the swarms suggested that it might not be necessary to use a large quantity of insecticide to kill the mosquitoes and stop the mating, unlike broadcast spraying. The mean height of swarms was 2 m and suggested also that swarms could easily be sprayed with appropriate pressure using hand sprayers.

The results of the CRT power simulations suggests that an intervention resulting in an 81% reduction in mosquito densities could be achieved with the number of villages as low as two (2 controls and 2 intervention villages); this would assume that the number of swarms comparable to the baseline sampling could be collected. Notably, an 81% reduction in mosquito density was recorded during a preliminary study in village VK5 in the western region of Burkina Faso [31]. On the other hand, if less than 40% reduction was expected, as many as 10 villages would be needed. Given the high impact of swarm killing observed in the preliminary study, it could be conservatively assumed that a 60% average reduction in mosquito densities could be achieved. With such effect size, the high mean number of swarms collected provided statistical power equivalent to 0.8 or higher (Fig. 6a). Adequate power could also be achieved for biting rate measured through HLC, assuming intervention impacts equivalent to 70% population reduction or higher, or alternatively through planning a larger number of HLC nights. It would be anticipated that the planned small-scale preliminary intervention prior to the full intervention would provide further data to confirm these estimated sample sizes.

The species composition of malaria vectors in the villages belonged only to the *An. gambiae* complex (*An. coluzzii*, *An. gambiae*, *An. arabiensis*), the main malaria vectors in Burkina Faso [18, 24, 40] and West Africa [41–43]. This would be consistent with the expectations in this ecological zone, as the type of larval sites (temporary, sunny, shallow water collections) found in the Sahelian climate associated with human activities (vegetable cultivation) favour the development of *An. gambiae* complex species. These three malaria vector species were found in indoor collections and in the swarms. These results showed that all malaria vectors in the study villages could be targeted by the intervention and that this strategy could be used to fight against all major malaria vectors in Burkina Faso because they all mate in the swarms [28, 30, 39, 44, 45].

In addition, the result showed, without surprise, that the female mosquito abundance in the indoor collection was twice as high as male abundance. This could be associated with the difference in feeding activities between the sexes. *Anopheles gambiae* abundance has varied between villages and was exceptionally higher in Nastenga, probably due to human activities (vegetable growing) that has led to profound changes in the habitat and the creation of new types of shelters favourable to the development of malaria vectors. The mean abundance of *An. gambiae* in both areas was extremely low (5 mosquitoes/house) compared to village VK5 (500 mosquitoes/house); the swarm intervention had a high impact [27]. Thus, the intervention could have a good impact in the study villages. Also, mosquito abundance was higher in August, September and October when compared to June, July, and November. This suggests that the intervention would be best carried out at the beginning of the rainy season when mosquito densities are low, in order to have a high impact and prevent the *An. gambiae* population increasing.

In this study, the HBR was higher indoors when compared to outdoors confirming that endophagy is usually the dominant behaviour in *An. gambiae* populations. It could be that the rate of indoor biting was higher because most of the human population stayed indoors for most of the night. Secondly, it could be that in these areas the *An. gambiae* populations would be resistant to the ITNs—this has been observed in other part of the same region [16, 17]. Results suggest that complementary tools would be needed to considerably reduce malaria transmission.

The mean proportion of the parity rate was very high in both areas and did not differ between the villages of each area, indicating that the populations studied were old. This age profile is associated with a high level of malaria transmission, which is probably due to the ineffectiveness of the current tools to control malaria vectors (ITNs, IRS). Mosquito longevity is a key parameter in malaria transmission. The planned intervention on swarms could help to reduce the proportion of old mosquitoes, as shown previously [27]. Like the parity rate, the insemination rate was high but did not vary significantly between the study sites. These two entomological parameters could be used to compare situations between villages to accurately assess the impact of the intervention, but a power analysis was not produced for these parameters, typically per village rates, because just one measurement per village does not give much statistical power compared to mosquito density sampling.

In general, the results showed that HBR and infection rates were different between the study sites, suggesting that the level of malaria transmission varied between the villages.

The intervention recently conducted in small scale in VK5 village has clearly affected the age structure of the male population, which was strongly shifted towards immature males and female insemination rate and female density [27]. Similarly, it is expected that the assessment of the impact of repeated target swarm killing with pesticide spray can result in a reduced mosquito density. This clearly indicates that targeting swarms offers an unrivalled opportunity to drastically reduce mosquito-borne pathogen transmission, specifically in places where residual malaria transmission persists despite high coverage by current intervention tools. As they all mate in swarms [28, 44, 46–48], this methodology can be used against all major malaria vectors in Africa.

## Conclusions

This study showed that the distribution of potential swarm markers and swarms in villages were clustered across space, making swarm-killing intervention easier. Power simulations showed that amongst the different entomological parameters proposed to assess the impact of planned swarm-killing intervention in two areas of western Burkina Faso, the direct sampling of swarms themselves provided the highest statistical power, thereby reducing the number of villages needed for a trial.

## Supplementary Information

**Additional file 1.** Location of the human dwelling compounds and swarms of *Anopheles gambiae* s.l. scattered through the villages in Area A (Santidougou, Kimidougou, Nastenga, Zeyama and Mogobasso).

**Additional file 2.** Location of the human dwelling compounds and swarms of *Anopheles gambiae* s.l. scattered through the villages in Area B (Synbekuy, Ramatoulaye, Syndombokuy, Lampa, Syndounkuy).

**Additional file 3: Table 1.** Composition of the Culicidae fauna in ten villages of the Areas A (Santidougou, Kimidougou, Nastenga, Zeyama and Mogobasso) and Area B (Synbekuy, Ramatoulaye, Syndombokuy, Lampa, Syndounkuy) over a period of six months from June to November 2016.

## Data Availability

The raw datasets are available from the corresponding author on reasonable request.

## Abbreviations

CRT: Cluster randomized trial
CSP: Circumsporozoite protein
EIR: Entomological inoculation rate
ELISA: Enzyme-linked immunosorbent assay
GPS: Global positioning system
HBR: Human biting rate
HLC: Human landing catches
IRS: Indoor residual spraying
ITN: Insecticide-treated net
IVCC: Innovative Vector Control Consortium
LLIN: Long-lasting insecticidal net
PCR: Polymerase chain reaction
PSC: Pyrethrum spray catch
VK5: Vallé du Kou (Village 5)
WHO: World Health Organization
WHOPES: World Health Organization Pesticide Evaluation Scheme
