# Supplementary figures and images for: Entomological baseline data collection and power analyses in preparation of a mosquito swarm-killing intervention in south-western Burkina Faso
Source: Malar J. 2021 Aug 23;20:346. doi: 10.1186/s12936-021-03877-x (PMC8381508; doi:10.1186/s12936-021-03877-x)

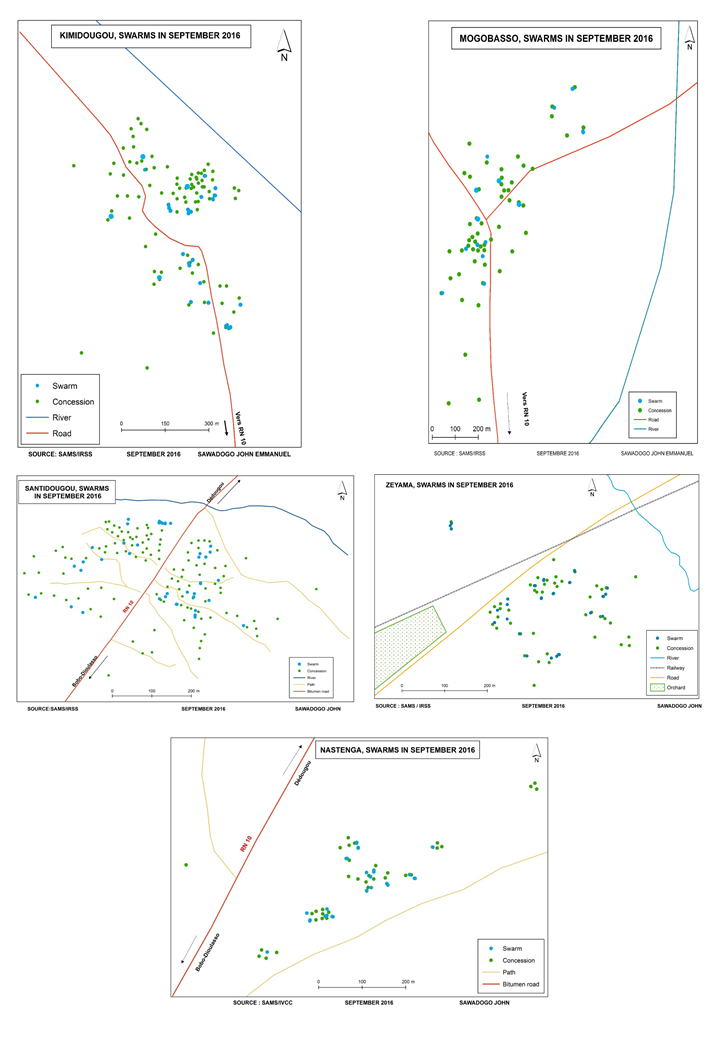

Supplement: Supplementary file 1 — Additional file 1. Location of the human dwelling compounds and swarms of Anopheles gambiae s.l. scattered through the villages in Area A (Santidougou, Kimidougou, Nastenga, Zeyama and Mogobasso). [file 12936_2021_3877_MOESM1_ESM.png]

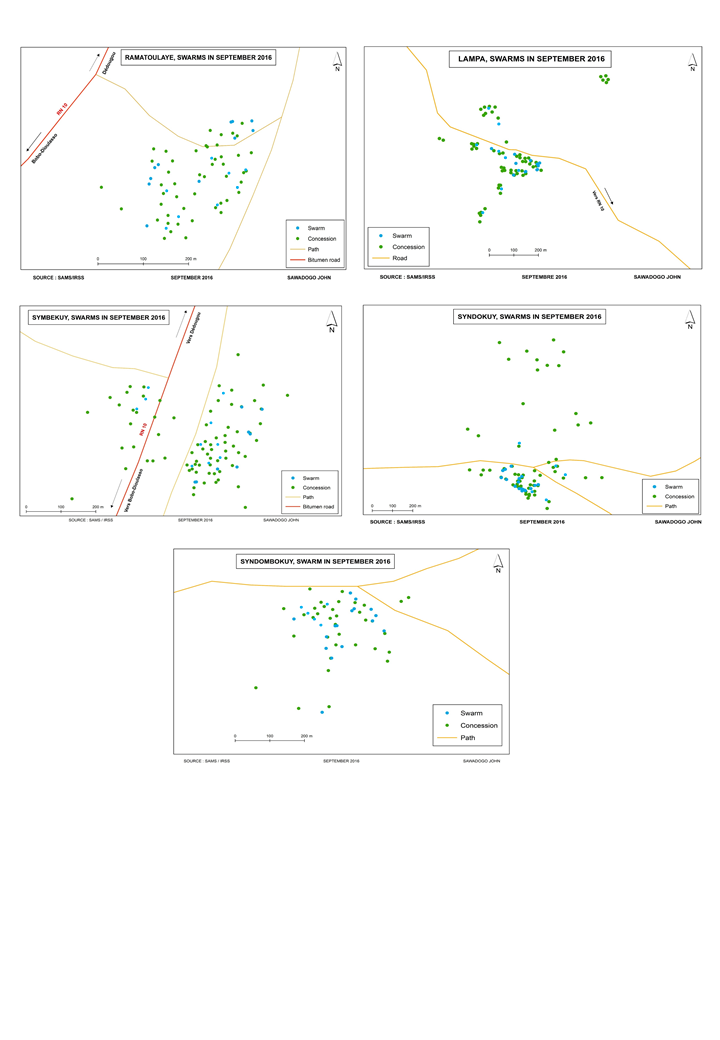

Supplement: Supplementary file 2 — Additional file 2. Location of the human dwelling compounds and swarms of Anopheles gambiae s.l. scattered through the villages in Area B (Synbekuy, Ramatoulaye, Syndombokuy, Lampa, Syndounkuy). [file 12936_2021_3877_MOESM2_ESM.png]
