# Supplementary material for: Entomological baseline data collection and power analyses in preparation of a mosquito swarm-killing intervention in south-western Burkina Faso
Source: Malar J. 2021 Aug 23;20:346. doi: 10.1186/s12936-021-03877-x (PMC8381508; doi:10.1186/s12936-021-03877-x)
Supplement: Supplementary file 3 — Additional file 3: Table 1. Composition of the Culicidae fauna in ten villages of the Areas A (Santidougou, Kimidougou, Nastenga, Zeyama and Mogobasso) and Area B (Synbekuy, Ramatoulaye, Syndombokuy, Lampa, Syndounkuy) over a period of six months from June to November 2016. [file 12936_2021_3877_MOESM3_ESM.docx]

Table 1. Composition of the Culicidae fauna in ten villages of the Areas A (Santidougou, Kimidougou, Nastenga, Zeyama and Mogobasso) and Area B (Synbekuy, Ramatoulaye, Syndombokuy, Lampa, Syndounkuy) over a period of six months from June to November 2016

| Area | Village | Month | Anophelinae | | Culicinae | | |
| --- | --- | --- | --- | --- | --- | --- | --- |
|  |  |  | *An. gambiae s.l.* | Other *Anopheles* | *Mansonia* | *Culex* | *Aedes* |
| Area A | Santidougou | June | 55 | 0 | 0 | 61 | 0 |
|  |  | July | 52 | 0 | 0 | 30 | 3 |
|  |  | August | 83 | 0 | 0 | 15 | 0 |
|  |  | September | 68 | 0 | 0 | 4 | 0 |
|  |  | October | 205 | 7 | 0 | 7 | 0 |
|  |  | November | 69 | 0 | 1 | 3 | 0 |
|  |  | **Total** | **532** | **7** | **1** | **120** | **3** |
|  | Kimidougou | June | 32 | 0 | 0 | 61 | 0 |
|  |  | July | 194 | 0 | 0 | 136 | 0 |
|  |  | August | 151 | 0 | 0 | 11 | 1 |
|  |  | September | 109 | 0 | 0 | 8 | 0 |
|  |  | October | 176 | 1 | 0 | 11 | 0 |
|  |  | November | 104 | 0 | 0 | 6 | 0 |
|  |  | **Total** | **766** | **1** | **0** | **233** | **1** |
|  | Nastenga | June | 90 | 0 | 0 | 2 | 0 |
|  |  | July | 308 | 7 | 1 | 26 | 1 |
|  |  | August | 162 | 0 | 0 | 60 | 0 |
|  |  | September | 317 | 0 | 0 | 10 | 0 |
|  |  | October | 418 | 27 | 0 | 16 | 0 |
|  |  | November | 125 | 4 | 0 | 28 | 0 |
|  |  | **Total** | **1,420** | **38** | **1** | **142** | **1** |
|  | Zeyama | June | 49 | 0 | 0 | 15 | 1 |
|  |  | July | 120 | 0 | 0 | 2 | 0 |
|  |  | August | 100 | 0 | 0 | 567 | 2 |
|  |  | September | 284 | 1 | 0 | 163 | 0 |
|  |  | October | 192 | 8 | 0 | 40 | 0 |
|  |  | November | 44 | 0 | 0 | 0 | 0 |
|  |  | **Total** | **789** | **9** | **0** | **787** | **3** |
|  | Mogobasso | June | 44 | 0 | 0 | 16 | 0 |
|  |  | July | 78 | 0 | 0 | 53 | 0 |
|  |  | August | 118 | 0 | 0 | 68 | 0 |
|  |  | September | 425 | 0 | 0 | 66 | 0 |
|  |  | October | 209 | 9 | 0 | 9 | 0 |
|  |  | November | 83 | 0 | 0 | 1 | 0 |
|  |  | **Total** | **957** | **9** | **0** | **213** | **0** |
| **Total in Area A** | |  | **4,464** | **64** | **2** | **1,495** | **8** |
| Area B | Synbekuy | June | 30 | 0 | 0 | 120 | 0 |
|  |  | July | 181 | 1 | 0 | 92 | 0 |
|  |  | August | 184 | 0 | 0 | 462 | 0 |
|  |  | September | 98 | 0 | 0 | 78 | 0 |
|  |  | October | 268 | 1 | 0 | 173 | 0 |
|  |  | November | 32 | 0 | 0 | 11 | 0 |
|  |  | **Total** | **793** | **2** | **0** | **936** | **0** |
|  | Ramatoulaye | June | 10 | 14 | 0 | 13 | 2 |
|  |  | July | 44 | 1 | 0 | 171 | 0 |
|  |  | August | 143 | 0 | 0 | 339 | 0 |
|  |  | September | 154 | 0 | 0 | 500 | 0 |
|  |  | October | 92 | 0 | 0 | 470 | 0 |
|  |  | November | 9 | 0 | 0 | 930 | 0 |
|  |  | **Total** | **452** | **15** | **0** | **2,423** | **2** |
|  | Syndombokuy | June | 77 | 0 | 0 | 111 | 3 |
|  |  | July | 130 | 0 | 0 | 49 | 0 |
|  |  | August | 407 | 0 | 0 | 21 | 0 |
|  |  | September | 386 | 0 | 0 | 0 | 0 |
|  |  | October | 41 | 0 | 0 | 23 | 0 |
|  |  | November | 41 | 0 | 0 | 23 | 0 |
|  |  | **Total** | **1,082** | **0** | **0** | **227** | **3** |
|  | Lampa | June | 10 | 0 | 0 | 10 | 0 |
|  |  | July | 241 | 0 | 0 | 0 | 0 |
|  |  | August | 472 | 0 | 0 | 613 | 0 |
|  |  | September | 120 | 0 | 0 | 763 | 1 |
|  |  | October | 159 | 11 | 0 | 784 | 0 |
|  |  | November | 9 | 3 | 0 | 523 | 0 |
|  |  | **Total** | **1,011** | **14** | **0** | **2,693** | **1** |
|  | Syndounkuy | June | 52 | 0 | 0 | 14 | 0 |
|  |  | July | 87 | 0 | 0 | 20 | 0 |
|  |  | August | 99 | 0 | 0 | 15 | 0 |
|  |  | September | 103 | 0 | 0 | 98 | 0 |
|  |  | October | 128 | 1 | 0 | 57 | 0 |
|  |  | November | 10 | 0 | 0 | 70 | 0 |
|  |  | **Total** | **479** | **1** | **0** | **274** | **0** |
| **Total in Area B** | |  | **3,817** | **32** | **0** | **6,553** | **6** |
| **Grand Total** | |  | **8,281** | **96** | **2** | **8,048** | **14** |
